# Land use alters arbuscular mycorrhizal fungal communities and their potential role in carbon sequestration on the Tibetan Plateau

**DOI:** 10.1038/s41598-017-03248-0

**Published:** 2017-06-08

**Authors:** Meng Xu, Xiaoliang Li, Xiaobu Cai, Xiaolin Li, Peter Christie, Junling Zhang

**Affiliations:** 1 Centre for Resources, Environment and Food Security, College of Resources and Environmental Sciences, China Agricultural University; Key Laboratory of Plant-Soil Interactions, Ministry of Education, Beijing, 100193 China; 2 Tropical Crops Genetic Resources Institute, Chinese Academy of Tropical Agricultural Sciences/Key Laboratory of Crop Gene Resources and Germplasm Enhancement in Southern China, Ministry of Agriculture, P.R. China, Danzhou, 571700 Hainan China; 3 Tibet Agricultural and Animal Husbandry College, Tibet University, Linzhi, 860000 China; 4 Key Laboratory of Ecosystem Network Observation and Modeling, Institute of Geographic Sciences and Natural Resources Research, Chinese Academy of Sciences, Beijing, 100101 China

## Abstract

Loss of belowground biodiversity by land-use change can have a great impact on ecosystem functions, yet appropriate investigations remain rare in high-elevation Tibetan ecosystems. We compared arbuscular mycorrhizal (AM) fungal communities in arable soils with those in native forest and grassland in southeast Tibet and investigated their potential contribution to carbon sequestration. The AM fungi were abundant and diverse. AM fungal diversity was significantly higher in grassland than in forest or arable land. Significant differences in AM fungal community composition were found among different land use types. The relative abundance of operational taxonomic units (OTUs) in forest and grassland were positively related to glomalin-related soil protein (GRSP), soil organic carbon, macroaggregates, and the unprotected and physically protected carbon, while the AM fungal community in arable soils was dominated by a few OTUs which were positively linked to soil pH. Changes in GRSP content were closely related to water-stable macroaggregates and carbon storage in grassland and forest soils but not in arable soil. Given the inevitable trend toward agricultural management this study emphasizes the need to implement effective agricultural practices that can enhance AM fungal activity to maintain soil quality and carbon sequestration for the sustainable development of this fragile ecosystem.

## Introduction

Soil is the largest organic carbon (C) reservoir in the terrestrial biosphere. It contains more than 1.5 trillion tonnes of C, roughly three times the C contained in all the vegetation worldwide and twice the amount of C stored in the atmosphere as CO_2_^1^. Hence, relatively minor changes in soil organic carbon (SOC) storage will result in substantial alterations in atmospheric CO_2_ concentrations. The Tibetan Plateau is the largest and highest plateau in the world with an average altitude of 4000 m above sea level (a.s.l.) and it contains ca. 33.5 Pg C in the top 75 cm of the soil profile, accounting for 2.5% of global soil C storage^2^. Given the vast storage of C in alpine ecosystems and their high sensitivity to climate change and anthropogenic activities, changes in soil C in high-elevation alpine ecosystems are of great concern, yet information on soil C changes and the fate of this enormous soil C stock remain uncertain. A regional scale survey suggests that topsoil carbon stocks in the Tibetan grasslands from 1980 to 2004 were not vulnerable to climate change^3^. However, other studies indicate that 1.01 Pg SOC has been lost in Tibet from the 1980s to the 2000s^4^. This seemingly contradictory evidence calls for a deeper mechanistic understanding of the accumulation and stability of soil C in alpine ecosystems.

Land-use change, in particular the conversion of natural vegetation to agricultural ecosystems, is regarded as one of the large sources of soil C losses^5, 6^. There is no exception to this principle in China^4, 7^ including the Tibetan Plateau. Several studies have shown that soil C status is impacted by land management^8^, land degradation^9^ and grazing intensity^10^. Recent research conducted at Haibei Alpine Meadow Ecosystem Station, Qinghai Province, reported a loss of SOC stock of 15 Mg C ha^−1^ (about 10.1% of total SOC) ten years after conversion from *Kobresia* pasture to arable land^11^. Land-use change is also a major threat to biodiversity and ecosystem services in addition to C loss. Land use can impose major influences on belowground soil biota, leading to changes in microbial communities and activities^12, 13^ or species loss^14^ which may have great impacts on ecosystem processes and properties including C cycling. Hence, it is suggested that the maintenance of appropriate SOC content and soil microbial biomass is essential for sustainable productivity, profitability and carbon sequestration^15^.

Soil microbes are directly responsible for the decomposition and the turnover of organic matter^16^ and contribute to soil C sequestration due to the formation and degradation of microbial byproducts, and/or indirectly affect C stability by increasing soil aggregation^17^. Arbuscular mycorrhizal (AM) fungi are an important group of soil microbes that are involved in carbon cycling. AM fungi can form potentially symbiotic associations with the majority of vascular plant species^18^. It is widely acknowledged that AM fungi can increase plant productivity^19^ and enhance host plant resistance to biotic and abiotic stresses^18^. In terms of C cycling, AM fungi are intimately involved in mediating C translocation from host plants to their hyphae and the soil matrix^20^ at both individual (e.g. increasing host biomass) and community (increasing plant productivity) levels. The extraradical mycorrhizal hyphae (EMH) comprise 20–30% of the soil microbial biomass^21^. In addition, EMH have been shown to affect soil structure and stabilize soils^22^, or they may act as a long-term binding agent through the production of glomalin (quantified from soil samples as glomalin-related soil protein, GRSP), a structural component of hyphae and spore walls which is released after decomposition^23^. Glomalin deposition contributes on average 5–10% of SOC. This has been suggested to be an important mechanism mediating soil C sequestration^23, 24^.

AM fungi are vulnerable to land-use change. An increase in land use intensity is often accompanied by a decrease in AM fungal species diversity^25, 26^ or EMH^27^, although studies also indicate that AM fungi have some resilience to disturbance^28, 29^. Only a limited number of studies have focussed on the influence of land use on AM fungi in China, and these have been restricted to AM fungi associated with a few specific plant species in small regions^30^. One larger scale study in the farming-pastoral ecotone of north China suggests that both available P and soil texture were significantly correlated with EMH and AM fungal richness and community composition^27^. AM fungi promote the sustainability of ecosystems by improving soil structure^31^. A previous field study in a tall prairie indicates that loss of AM hyphal abundance led to a concomitant cost in soil aggregation for which no other processes compensated^22^. Hence, land use-induced belowground changes in AM fungal community and abundance may be of great concern with respect to ecosystem function.

AM fungi are abundant on the Tibetan Plateau and some novel taxa are habitat specific^32^. As high-elevation ecosystems are increasingly threatened by intensified human perturbance and climate change, the alteration of AM fungal diversity and community structure due to land use is expected to have profound long-term effects on C sequestration. However, to date no information on the effect of land use on AM fungi is available in this fragile ecosystem. In the present study, therefore, we collected soil samples from three land use types (forest, arable land and grassland) in the southeast of the Tibetan Plateau where SOC in the arable land declined rapidly as a result of several decades of traditional cropping systems. This study aimed to investigate the effects of land use on AM fungal diversity and community composition and the relationship between AM fungi and soil aggregation. We also attempted to understand whether the alteration in AM fungal community affects soil C pools that are divided by different stabilization mechanisms (i.e., unprotected C, physically protected C, chemically protected C and biochemically protected C) based on the conceptual model proposed by Six *et al*.^33^. Soil C pools with different stabilities are important indices determining the turnover rates of SOC and soil C sequestration^34^. The present study aimed to provide a novel insight into the impact of anthropogenic influence on AM fungal diversity on the Tibetan Plateau and its potential ecological function in this vast, yet fragile, region.

## Results

### Land use impacts on soil properties and SOC pools

There were significant differences in soil physico-chemical properties among land use types (Table 1 and Supplementary Table S2). Across all three sampling sites soil pH had on average the highest value in arable land, with lower values in forest and grassland (Table 1). Available phosphorus (AP) content showed a similar trend. In contrast, soil total nitrogen (TN), total carbon (TC), soil organic matter (SOM), C:N ratio and available nitrogen (AN) in forest and grassland were generally higher than in cropped soils (Supplementary Table S2). The proportion of macroaggregates was significantly lower in arable land than in grassland or forest, while the microaggregate proportion showed the opposite trend. SOC storage in soils of arable land (11.3 g kg^−1^ soil) accounted for 37.8% and 44.3% of that in forest (29.9 g kg^−1^ soil) or grassland (25.5 g kg^−1^ soil) (Table 1). Across the three sites the unprotected C pool was the major fraction of SOC in forest and grassland but was significantly lower in arable land. The physically protected C pool showed a similar trend. In contrast, the concentration of chemically protected C did not differ significantly among the three land use types. The biochemically protected C pool had the lowest values among the SOC fractions, and the variation was site dependent, with significant differences among land use types at Sites 1 and 3 (Supplementary Table S3).

**Table 1:** Chemical properties, proportions of water-stable aggregates, SOC and N stock and four SOC fractions in soils of different land use types. Data are mean ± SE (n = 15). Means followed by the same letter do not differ significantly at *P* ≤ 0.05 by Duncan’s multiple range test.

Land use type	Forest	Grassland	Arable land
Soil property			
pH	5.41 ± 0.06 c	6.01 ± 0.05 b	7.53 ± 0.07 a
TC (g kg^−1^ soil)	33.23 ± 3.93 a	28.82 ± 3.25 a	13.94 ± 0.65 b
TN (g kg^−1^ soil)	2.54 ± 0.31 a	2.51 ± 0.23 a	1.53 ± 0.08 b
C:N	13.21 ± 0.33 a	11.39 ± 0.60 b	9.20 ± 0.30 c
SOM (%)	5.53 ± 0.49 a	5.47 ± 0.55 a	2.91 ± 0.45 b
AP (mg kg^−1^ soil)	13.66 ± 1.74 ab	11.86 ± 1.75 b	18.13 ± 1.90 a
AN (mg kg^−1^ soil)	10.76 ± 1.37 a	8.53 ± 1.36 a	3.16 ± 0.66 b
Water-stable aggregates (%)			
Macroaggregates (250–2000 μm)	69.97 ± 1.78 a	63.46 ± 2.40 b	44.56 ± 2.01 c
Microaggregates (20–250 μm)	25.57 ± 1.89 c	33.01 ± 2.34 b	50.51 ± 2.08 a
SOC fractions (g kg^−1^ soil)			
Unprotected C	16.6 ± 2.52 a	11.3 ± 2.16 a	2.3 ± 0.24 b
Physically protected C	6.3 ± 0.96 a	6.1 ± 0.63 a	1.7 ± 0.16 b
Chemically protected C	6.3 ± 0.78 a	7.3 ± 0.74 a	6.8 ± 0.48 a
Biochemically protected C	0.7 ± 0.06 ab	0.8 ± 0.08 a	0.5 ± 0.04 b

### Diversity and abundance of the AM fungal community

A total of 273,062 valid sequences with a length ≥ 300 bp were obtained from soil samples. Of these, 230,421 sequences belonged to Glomeromycota and 42,641 to non-Glomeromycota (i.e. 15.62% of the total). After trimming of the low quality sequences, 229,403 high quality sequences were used for subsequent analysis. The sequence number of each sample ranged from 2275 to 9583 (Supplementary Fig. S1). These sequences were assigned to a total of 84 AM fungal operational taxonomic units (OTUs) based on the BLAST hits in NCBI and examination of the NJ-tree with 97% similarity (Supplementary Fig. S2). The observed OTUs covered nine AM fungal families, namely Glomeraceae (average relative abundance: 66.14%), Gigasporaceae (15.54%), Acaulosporaceae (13.41%), Diversisporaceae (2.84%), Claroideoglomeraceae (0.76%), Archaeosporaceae (0.81%), Ambisporaceae (0.08%), Paraglomeraceae (0.02%) and Pacisporaceae (0.01%). Two OTUs which could not be assigned to known families were defined as putative new taxa (0.39%).

Land use type (but not sampling site) had a strong impact on AM fungal diversity indices (Supplementary Table S4). Irrespective of sampling site the average diversity indices of AM fungi (richness, Shannon-Wiener index, Simpson index and evenness) in grassland were significantly higher than in forest or arable land, while the latter two did not differ significantly from each other (Table 2). Both the abundance of AM fungi (hyphal length density, HLD) and total GRSP concentrations (T-GRSP) and easily-extractable GRSP (EE-GRSP) were significantly lower in cropped soil compared to forest and grassland soils (Table 2). However, within each site HLD did not differ significantly among the three land use types (except at Site 2). Compared to grassland, EE-GRSP in forest varied with site but was generally significantly higher than in arable land (Supplementary Table S4). T-GRSP concentrations were highest in grassland and did not differ significantly between forest and arable land (except at Site 1).

**Table 2:** Diversity indices of AM fungal community, hyphal length density (HLD) and glomalin content in soils of three different land use types. Data are mean ± SE (n = 15). Means followed by the same letter do not differ significantly at *P* ≤ 0.05 by Duncan’s multiple range test.

Land use type	Forest	Grassland	Arable land
AM fungal community diversity			
Richness	22.40 ± 1.34 b	32.00 ± 2.24 a	23.87 ± 1.93 b
Shannon-Wiener index	1.58 ± 0.14 b	2.21 ± 0.11 a	1.59 ± 0.14 b
Simpson	0.66 ± 0.05 b	0.82 ± 0.02 a	0.62 ± 0.05 b
Evenness	0.51 ± 0.04 b	0.64 ± 0.02 a	0.50 ± 0.04 b
HLD (m g^-1^ soil)	38.44 ± 3.96 a	35.60 ± 5.30 a	19.57 ± 2.00 b
Glomalin content (mg g^−1^ soil)			
T-GRSP	3.65 ± 0.24 b	4.51 ± 0.22 a	2.27 ± 0.05 c
EE-GRSP	2.10 ± 0.10 a	2.14 ± 0.11 a	1.07 ± 0.04 b

### Composition of the AM fungal community

**Figure 1:**
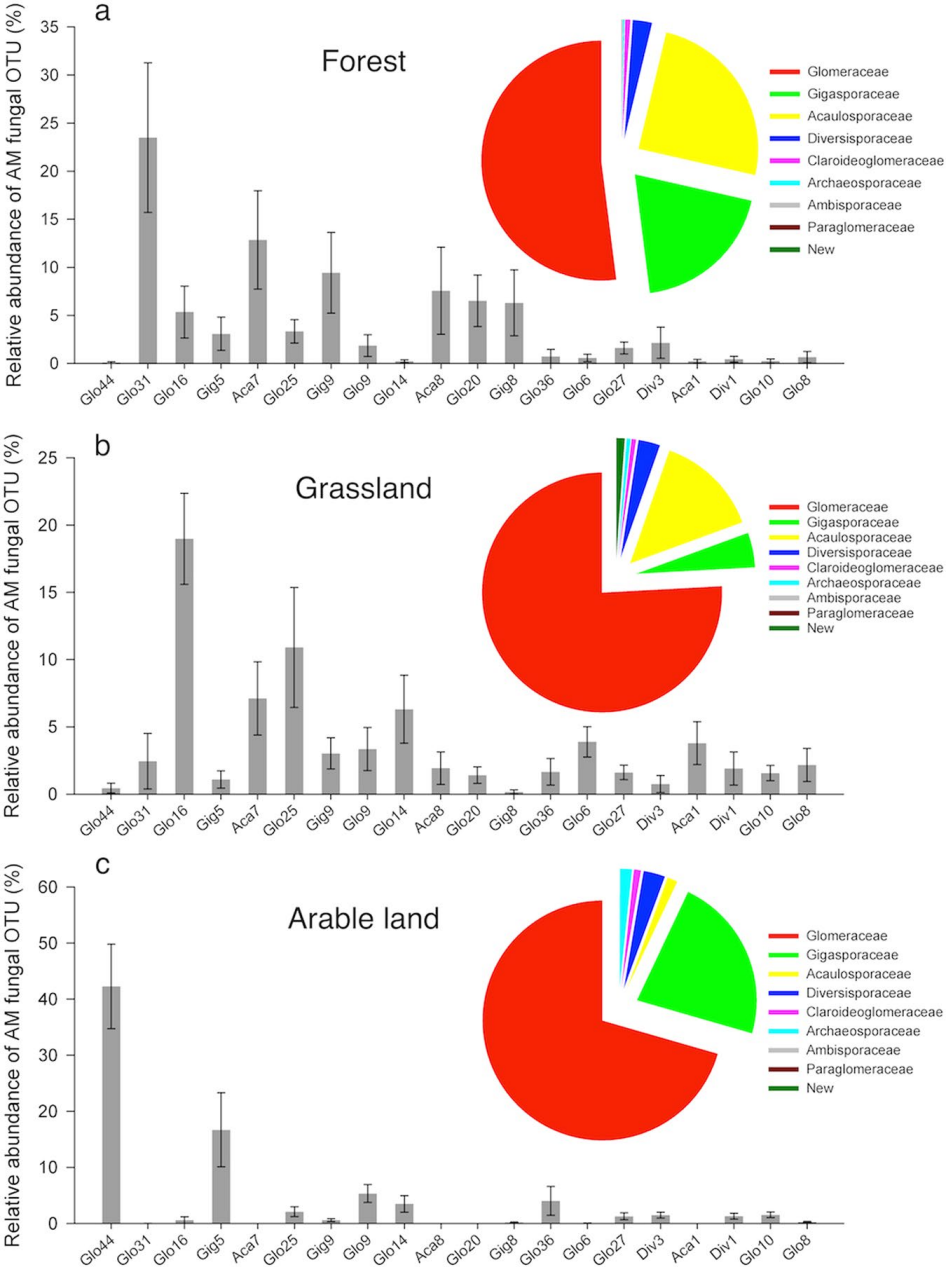
Relative abundance (%) of AM fungal operational taxonomic units (OTUs) in soils sampled from three land use types. The relative abundance of AM fungal OTUs grouped by families, and the relative abundance of 20 dominant AM fungal OTUs (average relative abundance across all samples ≥ 1.1%) in (**a**) forest, (**b**) grassland and (**c**) arable land.

**Figure 2:**
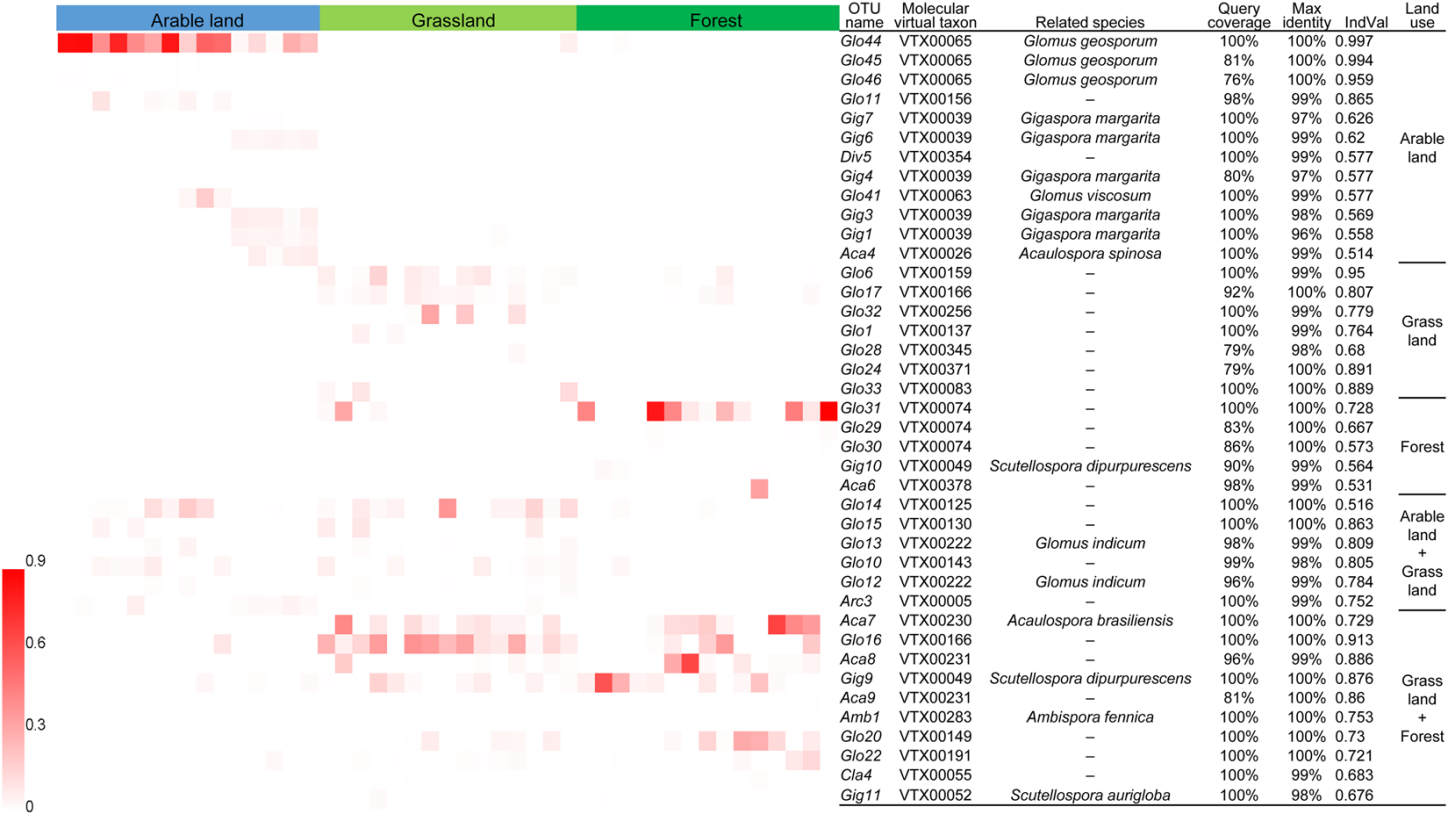
Heatmap of relative abundances of AM fungal indicator species that were found to differ significantly among arable lands, grasslands and forests. The 45 columns represent samples in arable lands (15 columns), grasslands (15 columns) and forests (15 columns). The ranked OTUs were indicator species of each land-use type and two land-use types combined (arable lands and grasslands or arable lands and forests).

Land use had a strong impact on the AM fungal community by influencing the proportion of the three dominant AM fungal families. Glomeraceae were substantially lower in forest than in grassland and arable fields. Gigasporaceae were less abundant in grassland compared to arable fields and forest, and Acaulosporaceae in arable land comprised a minor proportion compared to forest and grassland (Fig. 1). The AM fungal community in arable land was dominated by a few OTUs of which Glo44 was predominant, followed by Gig 5, and the remainder were less abundant (Fig. 1c). The OTUs in forest were dominated by Glo31, Aca7, Gig9, Aca8, Glo20 and Gig8 (Fig. 1a), and the remaining OTUs in grassland were relatively homogeneously distributed (Fig. 1b). Out of the total of 84 OTUs, 24 OTUs of AM fungi were found to be indicator species of arable land (12), grasslands (7), or forests (5) (Fig. 2). We also observed 6 OTUs from both arable land and grasslands, and 10 OTUs from arable land and forests.

NMDS ordination revealed significant differences in AM fungal community composition among the three land use types (Supplementary Fig. S3). Differences in community structure were significantly correlated with soil pH, total C, available N, C:N ratio and available P. Variation partition analysis shows that approximately 45.1% of the variation in AM fungal community composition was explained uniquely by land use type, sampling site, soil properties and their joint effects (Supplementary Fig. S4). Soil physico-chemical properties and land use type were the main factors and their joint effects explained 20.5% of the variation.

### Relationship between AM fungal factors and C storage

**Figure 3:**
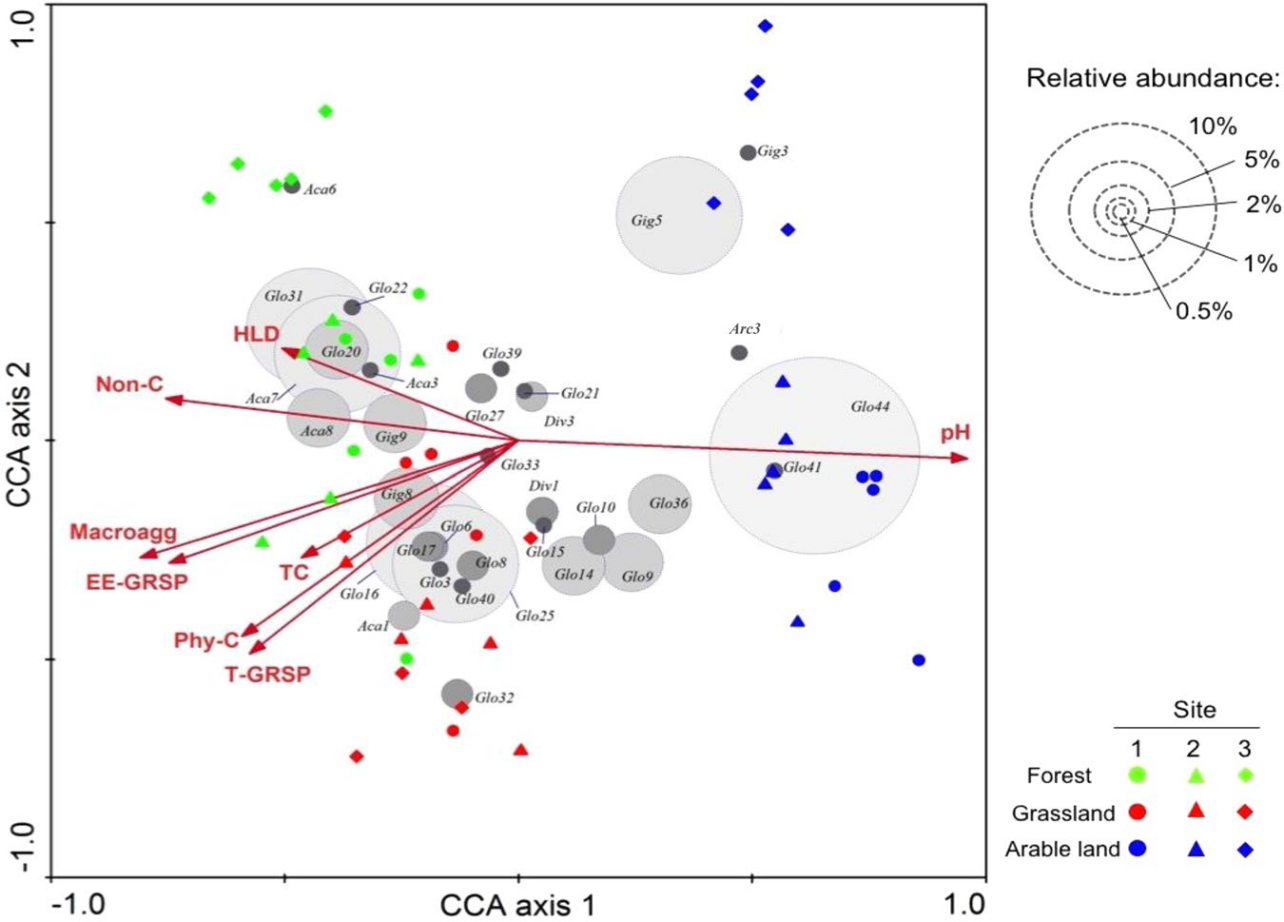
Sample and species plot derived from the canonical correspondence analysis (CCA) of AM fungal communities in soils sampled from three land-use types. The CCA is based on all AM fungal OTUs (84) but only the 34 OTUs that on average represented at least 0.5% of the total amplicon are shown. The relative abundance of each OTU is indicated by dot size. The environmental variables explained 10.9% and 7.1% of the variations in the first two axes. HLD, hyphal length density; T-GRSP, total glomalin related soil protein; EE-GRSP, easily-extractable glomalin related soil protein; Non-C, unprotected C; Phy-C, physically protected C; Macroagg, macroaggregates.

**Figure 4:**
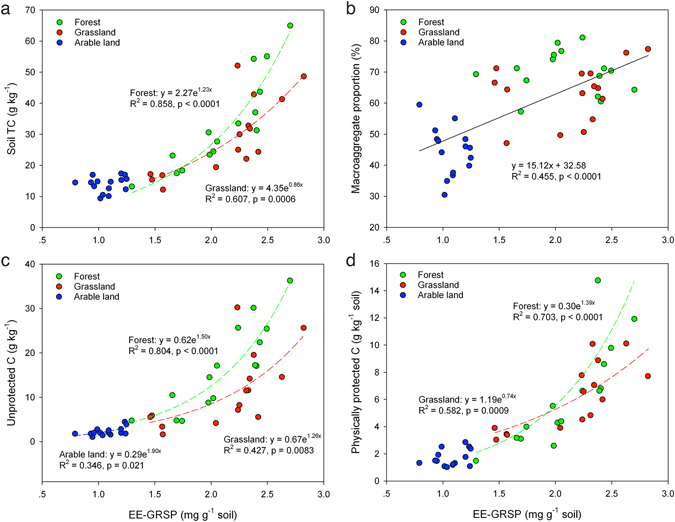
Relationships between EE-GRSP and soil C storage and soil aggregation. Regression analyses showing the relationship between EE-GRSP and (**a**) soil TC, (**b**) proportion of macroaggregates, and (**c**) C concentrations in the unprotected C fraction and (**d**) physically protected C fraction. In a, c and d, the dashed line with the same color of symbols indicates the predicted relationship between EE-GRSP and soil C storage for each land use type. In b, the solid line indicates the predicted relationship between EE-GRSP with soil aggregation across all three land use types.

CCA analysis shows that the relative abundance of dominant AM fungal OTUs in forest and grassland were positively related to EE-GRSP, T-GRSP, SOC, macroaggregates, and the unprotected and physically protected C fractions (Fig. 3). However, the abundance of several OTUs in arable land was positively linked to soil pH. The abundance of EMH and glomalin content were both significantly correlated with macro- and microaggregate distribution across all sampling sites, and these parameters were correlated with the soil C stock and the two C pool fractions (Supplementary Table S5). However, most of these correlations remained highly significant across all sampling sites in grassland and forest, but became insignificant or marginally significant in arable land (Supplementary Table S5). Regression analysis shows that soil TC and two major C pools (unprotected and physically-protected C) increased exponentially with increasing glomalin concentration in forest and grassland (Fig. 4). In arable land, however, there was no significant, or only a weak, relationship between glomalin concentration and soil C stabilization.

## Discussion

Our study is the first to investigate the AM fungal community as affected by land use on the Tibetan Plateau. Our results are similar to those of other studies^25–27^, showing higher averaged AM fungal diversity in grasslands than in forests or arable land (Table 2). This may be associated with the relatively high plant diversity in grassland, and diverse plant species provide more niches hosting AM fungi. An early study conducted in grassland showed a positive relationship between plant diversity and AM fungal diversity^35^. In addition, perennial herbs such as *Potentilla* spp.*, Carum carvi*, *Gueldenstaedtia*, and *Duchesnea indica* can form dense root mats and consistently invest large proportions of C in the roots and also likely host diverse AM fungi. Furthermore, the relatively lower AP content in grassland soil (Table 1) may also promote AM fungal diversity. Within each sampling site the diversity index was higher in grassland while the significant effect was site-specific (Supplementary Table S4). The relatively lower diversity of AM fungi in forests compared to grassland is in accordance with other studies in temperate forests^36^ and worldwide^37^. Trees are often associated with ectomycorrhizal fungi and forest soils are rich in decomposing fungal species, and consequently the richness of AM fungi may be low. The average AM fungal diversity of arable land was more comparable to that of forest (Supplementary Table S4). This result contrasts with the general conclusion of negative correlation between land use intensity and AM fungal richness in soils^25, 27^ and roots^38^, but is consistent with a recent study conducted in a range of habitats in Estonia comparing forested and structurally open habitats with a gradient of land use intensity^39^. Agriculture in the local region is characterized by low inputs and less intensive management, which is reflected by the relatively low nutrient concentrations, in particular available P concentrations. Local farmers are not in pursuit of high grain yields which are used mainly for brewing. Arable land is not intensively managed, e.g. no/few applications of inorganic fertilizers and infrequent applications of yak dung, and with reduced tillage or no tillage as compared with intensive agriculture in other regions of China. In addition, the occurrence of weeds increases plant diversity in the arable fields and may also act as hosts for AMF. Finally, arable land accounts for only a small proportion of a landscape that is dominated by forests and grasslands. The adjacent forests and grasslands may also act as reservoirs to sustain expansion and colonization by AM fungi in the arable land.

In contrast to AM fungal diversity, the AM fungal community exhibited different structures among the three land use types investigated (Fig. 1 and Supplementary Fig. S3). Similar results have been obtained in numerous other studies^27, 40^. Changes in vegetation, soil pH^25, 39^, available P^27^, soil type^25^ and intensity of soil disturbance may account for the community change. Our results show that, in addition to biotic factors, soil pH, total N and C, and AP were the major abiotic variables affecting community structure (Supplementary Figs S3 and S4). In the present study it was not possible to distinguish the separate contributions of biotic and abiotic factors to community change. However, joint effects between land use types and soil factors accounted for a large proportion of community variation (Supplementary Fig. S4). The AM fungal community in arable land was dominated by Glomeraceae (in particular Glo44, *Funneliformis caledonium*; Fig. 1c). This is consistent with the notion that arable farming generally favors AM fungi within this family^41^. The indicator OTU Glo44 is highly affiliated with VTX00065 (Maarj*AM* database), which is widely distributed in various ecosystems often characterized by high soil pH (>7)^42, 43^. In addition to the dominance of Glo20 and Glo31, prevalent OTUs in forest were Acaulosporaceae (Aca7, *Acaulospora brasiliensis*) and Gigasporaceae (Gig8 in Site 1, *Gigaspora rosea*) and Gig9 (*Scutellospora dipurpurescens*) (Fig. 1a). In contrast, plant species richness in grassland may provide broad niches for more diverse AM fungi, as the AM fungal community in grassland is more homogeneously distributed, except for the three dominant OTUs from the Glomeraceae (Glo16 and Glo25) and Aca7 (Fig. 1b). Relatively high abundance of Acaulosporaceae has been observed in high-elevation regions with harsh climatic conditions compared with temperate systems^44^, and may correspond to low pH environments^45^. In the present study Gigasporaceae were prevalent in forest and arable land. Similar results have been reported in roots of *Polylepis australis* from forest on the high mountains of central Argentina^46^ and arable land^47^. Compared with other phylogenetic groups, members of the Gigasporaceae invest more biomass in extraradical hyphae than in root-borne structures^48^. Several isotope tracer studies have also provided direct evidence that members of the Gigasporaceae are stronger sinks for plant C than other lineages^49^.

We found that dominant AM fungal OTUs detected in grassland and forest were positively correlated with HLD, GRSP, SOC and the two C pools, while OTUs in arable land were correlated with soil pH (Fig. 3). It is generally acknowledged that soil pH acts as a strong filter on AM fungi ^25, 39^. In the present study it is interesting to find that a positive correlation between HLD/GRSP and soil C was found only in grassland and forest, but not in arable land (Supplementary Table S5). In addition, we found strong positive correlations between GRSP and the cPOM-C (coarse non-protected particulate organic matter-C) fraction (unprotected C) and the microaggregate-associated C fraction (physically stabilized C). Cultivation consistently reduced the abundance of AM hyphae and glomalin concentrations at three sampling sites, and on average HLD and glomalin (T-GRSP and EE-GRSP) in arable land decreased by nearly 50% when compared to grassland and forest (Table 2). Our results indicate that the influence of EMH and GRSP on soil C storage is likely much stronger in natural habitats or less disrupted ecosystems than in arable land where physical disturbance or monoculture may have disrupted the extraradical hyphal network. Similar results were also reported from a long-term field experiment in a prairie field^22^ in which the authors show a linear reduction in SOC storage due to loss of AM hyphal network following six years of fungicide use. In contrast to their study, we found that the decline in soil C was more associated with the loss of GRSP across the land use gradient as shown by the exponential model (Fig. 4a) and by correlation analysis (Supplementary Table S5). Glomalin C was shown to represent up to 4–5% of total soil C in a tropical forest soil^50^. In addition, the hyphal mesh can facilitate the production of glomalin to contribute to soil aggregation. In the present study the higher abundance of Acaulosporaceae observed in forest and grassland soils (Fig. 1a and b) may be responsible for their higher GRSP contents, as pot experiments show that Acaulosporaceae species yielded the highest levels of glomalin followed by Gigasporaceae and Glomeraceae species^51^. In addition, the higher richness and diversity of plants and AM fungi may affect soil aggregation at different levels, e.g. at community, individual root system and mycelium levels^31^. As the AM fungal community varied greatly among the three land use types, whether or not community change reflects changes in functional traits and the underlying mechanisms by which AM fungi affect soil aggregation and soil C stocks warrant further investigation.

Southeast Tibet contains various vegetation types and may act as a potential C sink due to the large living biomass and productivity as a result of the high forest cover and slow decomposition rate due to low temperatures^52^. Based on the data of the current study, soil C stored in the top 20 cm soil of forest and grassland is estimated to be approximately 0.212 Pg in Nyingchi region (Supplementary Methods) with a higher SOC density of 7.23 kg m^−2^ than the vast Tibetan grassland (4.42 kg m^−2^ in top 30 cm)^53^. The area of land use change on the Qinghai-Tibetan Plateau is relatively small compared to the remainder of China^54^. Nevertheless, a slight change can lead to serious consequences, given the fact that C loss is mainly attributed to decreases in unprotected and physically protected C which may lead to negative feedback to climate change in these sensitive alpine ecosystems. Furthermore, changes in soil macroaggregates may have long-standing consequences for ecosystem properties such as soil porosity, gas exchange, water infiltration and erosion resistance^55^. As AM fungal community composition is fundamentally changed in arable land after long-term crop cultivation, changes in or losses of specific taxa may lead to the loss of some key ecological functions that may not be easily compensated. Furthermore, it remains unknown whether changes in the AM fungal community are reversible in the long run. While the prevalence of agricultural management is inevitable due to the increasing demand of the increasing population and economic development, it is therefore imperative to implement more effective agricultural practices to compensate losses in soil C for the sustainable development of this fragile ecosystem.

## Methods

### Site description and sampling

The study was conducted in Nyingchi region (26°52′-30°40′ N, 92°09′-98°47′ E) in the southeast part of the Tibetan Plateau. The average altitude is approximately 3000 m above sea level. The mean annual temperature (MAT) ranges from 7 to 16 °C and the mean annual precipitation (MAP) is 600 to 800 mm, about 92.4% of which is distributed from April to October. About 46% of the land area is covered by forest (2.64 × 10^6^ ha) which accounts for nearly 80% of the total forested area on the Tibetan Plateau. The area of grassland is 2.91 × 10^5^ ha of which 5.25 × 10^4^ ha is suitable for pasture. Nyingchi region is also one of the most important and historical areas for agriculture in Tibet. The typical traditional tillage system is fallow and mono-cropping of annual crops with low fertilizer inputs^56^. The main grain crops are winter wheat (*Triticum aestivum* L.) and hull-less barley (*Horderum vulgare* L. var. *nudum* Hook. f.) and the production of fruit and vegetables has also increased in recent years. The area of land currently under cultivation is about 1.87 × 10^4^ ha, and has expanded rapidly in recent years due to the increasing demand by the dense human population and the rapid development of the regional economy. According to the Statistics Department of Nyingchi Region, the cultivated area increased at a rate of about 1.4% per year from 2011 to 2013 and the rates of increase of the wheat and vegetable cultivation areas were 4% and 1.6% per year, respectively (from ‘the Statistical Bulletin of National Economic and Social Development of Nyingchi Region′, http://linzhinews.com/economy/qncj/).

Arable fields, grasslands and forests were selected from three sampling sites at least 20 km apart (Supplementary Table S1). The agricultural fields in these sites have been cultivated for more than 60 years. The arable fields at Sites 1 and 2 are cultivated with wheat and at Site 3 with hull-less barley. These arable fields have received low fertilizer inputs (household manure) and no herbicides. The native forests are temperate coniferous and broad-leaved mixed secondary forests. The grasslands were not managed and were slightly to moderately grazed. The dominant plant species in the forest and grassland at each site are shown in Supplementary Table S1 online.

Soil sampling was conducted in July 2012. Five quadrats (5 m × 5 m) approximate 20–30 m apart were randomly selected in each arable field for soil sampling. At each quadrat three soil monoliths (20 cm × 20 cm) of topsoil (0–20 cm) were randomly collected and combined to give one composite sample from each of the five replicate quadrats. Soil samples were also collected from adjacent forest and grassland (within a distance of 500 m) using the same sampling strategy to ensure that the soil had similar soil texture. A total of 45 soil samples (3 land use types × 5 replicates × 3 sites) were obtained. The soil samples were placed in polyethylene bags, stored on ice and transported to the laboratory. The soil samples were sieved (<2 mm) to remove visible stones, animals, root fragments and plant material. Sub-samples of fresh soils were used for DNA extraction and N_min_ (NH_4_^+^-N and NO_3_^−^-N) extraction and stored at −80 °C and 4 °C, respectively. The remaining portions of the soil samples were air-dried and stored at room temperature for further analysis.

### Determination of soil physico-chemical properties

Soil pH was measured in 1 M KCl (soil:water ratio 1:2.5). SOM content was determined using 0.25 mm sieved soil by wet oxidation followed by titration with ferrous ammonium sulfate. Soil AP was extracted with 0.5 M NaHCO_3_ and then determined by colorimetry. For the determination of N_min_ (NH_4_^+^-N and NO_3_^−^-N), soil samples were extracted with a 1:10 suspension of soil in 0.01 M CaCl_2_ solution and analyzed using a continuous flow analyzer (TRAACS 2000, Bran and Luebbe, Norderstedt, Germany). Soil AN content was thus calculated as the sum of NH_4_^+^-N and NO_3_^−^-N. TC and TN in the soil were analyzed using an elemental analyzer (EA1108, Carlo Erba, Torino, Italy). Water-stable aggregates were divided into macroaggregates (250–2000 μm size fraction) and microaggregates (20–250 μm size fraction) and were measured following the method of Leake *et al*.^22^.

### Soil C pool fractionation

Various soil C pools were separated by a combination of physical and chemical fractionation techniques in a three-step process based on the conceptual SOC fraction model proposed by Six *et al*.^33^ and a detailed methodological description is given in Stewart *et al*.^57^. The separated SOC fractions are grouped into four C pools (unprotected, physically protected, chemically protected and biochemically protected) based on the assumed linkages between the isolated fractions and the protection mechanisms involved in the stabilization of organic C within that pool. The unprotected C pool consists of the cPOM fraction (>250 μm) and the LF fraction (fine non-protected POM). The physically protected C pool refers to the μagg fraction (microaggregate, 53–250 μm) as a whole and the iPOM fraction (microaggregate-protected POM). The chemically protected C pool corresponds to the H-dSilt and H-dClay fractions (hydrolysable easily dispersed silt and clay, < 53 μm) which is stabilized due to the chemical or physico-chemical binding between SOC and soil minerals (i.e. clay and silt particles). The biochemically protected C pool refers to the NH-dSilt and NH-dClay fractions (non-hydrolysable easily dispersed silt and clay) which is stabilized due to its own chemical composition (e.g. recalcitrant compounds such as lignin and polyphenols) and through chemical processes (e.g. condensation reactions) in soil^33^.

### Assessment of hyphal length density and extraction of soil glomalin

Extraradical hyphae were extracted from each soil sample and stained with Trypan blue and the HLD of AM fungi was determined using the grid line intersect method in which AM fungal hyphae were distinguished from non-AM hyphae by the presence of irregular septa, dichotomous branching, irregular wall thickness and/or connection to chlamydospores^58^. The extraction of total glomalin related soil protein (T-GRSP) was done following the method of Wright and Upadhyaya^59^. Protein content was determined by the Bradford dye binding assay with bovine serum albumin as the standard^60^.

### DNA extraction and 454 pyrosequencing

Soil DNA was extracted from 0.5 g samples using a Fast DNA SPIN Kit (MP Biomedicals LLC, Santa Ana, CA) following the instruction manual of the manufacturer. The quality and quantity of the extracted DNA samples were checked on a 1.0% (w/v) agarose gel and then stored at −20 °C for subsequent analysis.

All DNA samples were subjected to nested PCR. The first PCR reaction was performed with AML1 and AML2 to amplify about 795 bp fragment of SSU rDNA^61^. PCR reactions were carried out in a final volume of 25 mL with 2 μL 10 × PCR buffer, 0.2 mM dNTPs, 0.3 μL of each primer (25 μM stock), 1 U EasyTaq DNA Polymerase (TransGen Biotech, Beijing, China) and 1 μL DNA template. The PCR program was as follows: 94 °C for 5 min; 30 × (94 °C for 30 s; 60 °C for 45 s and 72 °C for 1 min); and 72 °C for 10 min; 12 °C for 10 min. Successful products of the first amplification were diluted to 1:100 and then used as a template in the second PCR with NS31^62^ and AM1^42^. This pair of primers was used to amplify Glomeromycota sequences in roots of *Trachycarpus fortune* using 454 sequencing^63^ and in other natural ecosystem studies^64^. We amplified Glomeromycota sequences using the Amplicon Fusion Primers 5′-A-x-NS31-3′ and 5′-B-AM1-3′, where A and B represent the pyrosequencing adaptors (CCATCTCATCCCTGCGTGTCTCCGACGACT and CCTATCCCCTGTGTGCCTTGGCAGTCGACT) and x represents a 10 bp-tag for sample identification. PCR reactions were run under the same conditions as described above.

The second PCR reactions were carried out in a final volume of 50 μL with 5 μL 10 × PCR buffer, 37.2 μL ddH_2_O, 0.2 mM dNTPs, 0.6 μL of each primer (25 μM stock) each primer, 3U EasyTaq DNA Polymerase (Trans Gen Biotech, Beijing, China) and 2 μL DNA template. The PCR program was as follows: 94 °C for 5 min; 25 × (94 °C for 30 s; 60 °C for 45 s and 72 °C for 1 min); and 72 °C for 10 min; 12 °C for 10 min. PCR products from each sample were purified with a PCR Purification Mini Kit (Aidlab Biotechnologies Co., Ltd, Beijing, China) according to the manufacturer’s instructions and then sent to Majorbio Pharm Technology Co., Ltd., (Shanghai 201203, China) for 454-pyrosequencing.

454-pyrosequencing was conducted using the 454 GS-FLX Titanium sequencing platform of Roche (Basel, Switzerland). Resulting sequence sets were subjected to the denoising and clustering pipeline of Quantitative Insights Into Microbial Ecology (QIIME) pipeline for 18 S dataset (http://qiime.sourceforge.net/tutorials/processing_18S_data.html). In brief, the 454-pyrosequencing reads with ambiguous nucleotides, a quality score < 20, lacking a complete barcode and NS31 primer, or shorter than 300 bp (excluding the barcode and primer sequences) were removed and excluded from further analysis. Chimeric reads were identified using Chimera Slayer^65^ and removed. Reads were trimmed and assigned into the same OTU using a 97% identity threshold and the most abundant sequence from each OTU was selected as a representative sequence for that OTU. All the OTUs with sequence numbers ≤ 5 were removed in subsequent analysis. Representative sequences from each OTU clade were blasted against the GenBank non-redundant nucleotide database to detect non-Glomeromycota sequences. We constructed a neighbor joining tree to further identify our OTU belonging to the family Glomeromycota. Representative sequences from each encountered AM fungal OTU have been deposited in GenBank (accession numbers KU167955-KU168037). We used the online database Maarj*AM* (http://maarjam.botany.ut.ee; status on 22 April, 2015)^64^ to compare the OTUs obtained in the present study with the AM fungal OTUs in other ecosystems. We first used one representative sequence per OTU to BLAST against the Maarj*AM* database and grouped our sequences into the corresponding molecular virtual taxa with sequence identity ≥ 97%. Virtual taxa corresponding to OTUs were re-searched to confirm which morphospecies were related to the virtual taxa using the “search by molecular virtual taxon” tool in the Maarj*AM* database.

### Statistical analysis

Sequencing and sampling efficacy were assessed with rarefaction analysis of data subsets using the ‘rarefy’ and ‘specaccum’ function from R package VEGAN^66^. Relative abundance of an OTU or family in a sample was calculated as the proportion of its sequence number in total sequences. The AM fungal community-related analyses were based on relative abundances of OTUs per sample. Two-way analysis of variance was used to examine the significance of land use type, sampling site and their interactive effects on the relative abundance of each AM fungal OTU and family, AM fungal diversity indices (richness, Shannon-Wiener index, Simpson index and evenness), HLD, glomalin content (EE-GRSP and T-GRSP), soil properties, aggregate proportions and SOC fractions. Mean values were compared using Duncan’s multiple range test. Spearman correlation analysis was used to test the relationship between T-GRSP, EE-GRSP, HLD, macroaggregates, microaggregates and C fractions in the soil. Regression analyses were performed to simulate the relationship between GRSP and soil C and aggregation. All the correlation and regression analyses and analysis of variance were performed using the SPSS 18.0 software package.

Nonmetric multidimensional scaling (NMDS) was used to determine how land use structured the AM fungal communities in the soil. The OTU data matrix was composed of the relative abundance of a particular AM fungal OTU in each sample, and the data were square root-transformed in order to downweigh the importance of abundant OTUs. All environmental variable data included in the NMDS plot were log_e_(x + 1) transformed and fitted as vectors onto the NMDS plot using the function ‘envfit’ from the vegan library. We adopted the function ‘ordiellipse’ from the ‘vegan’ library to test the difference in AM fungal communities under different land use types using the standard error of the average scores. The function ‘varpart’ from the ‘vegan’ library^66^ was used to partition the variation in AM fungal communities into three explanatory factors: soil properties (soil pH, TC, TN, C:N ratio, SOM, available P, available N and soil moisture), land use types and sampling sites. To determine AM fungal indicator species for each land use type we conducted indicator species analyses for the AM fungal community of each land use type using the function ‘multipatt’ from the ‘indicspecies’ library^67^. OTUs with IndVal values ≥ 0.5 and *P* ≤ 0.05 were recorded as indicator species for a particular treatment. All the analyses above were conducted in the R version 3.0.2.

Canonical correspondence analysis (CCA) was used to explore the relationships between AM fungal communities and HLD, GRSP, macroaggregates and C fractions using CANOCO 4.5 for Windows (Microcomputer Power Inc., Ithaca, NY). AM fungal OTU data were square root-transformed during the CCA procedure. Forward selection tests were conducted using 499 permutations and the Monte Carlo permutation test with *p* < 0.05 was used.

## Electronic supplementary material

Supplementary materials
