# Supplementary material for: Land use alters arbuscular mycorrhizal fungal communities and their potential role in carbon sequestration on the Tibetan Plateau
Source: Sci Rep. 2017 Jun 8;7:3067. doi: 10.1038/s41598-017-03248-0 (PMC5465222; doi:10.1038/s41598-017-03248-0)
Supplement: Supplementary file 1 — Supplementary materials [file 41598_2017_3248_MOESM1_ESM.doc]

Supplementary Information for

**Land use alters arbuscular mycorrhizal fungal communities and their potential role in** **carbon sequestration on the Tibetan Plateau**

MengXu^1,4^, Xiaoliang Li^1,2^, XiaobuCai^3^, Xiaolin Li^1^, Peter Christie^1^, Junling Zhang^1^, *

^1^ Centre for Resources, Environment and Food Security, College of Resources and Environmental Sciences, China Agricultural University; Key Laboratory of Plant-Soil Interactions, Ministry of Education, Beijing 100193, China

^2^ Tropical Crops Genetic Resources Institute, Chinese Academy of Tropical Agricultural Sciences / Key Laboratory of Crop Gene Resources and Germplasm Enhancement in Southern China, Chinese Ministry of Agriculturea, Danzhou 571700, Hainan, China

^3^ Tibet Agricultural and Animal Husbandry College, Tibet University, Linzhi 860000, China

^4^ Key Laboratory of Ecosystem Network Observation and Modeling, Institute of Geographic Sciences and Natural Resources Research, Chinese Academy of Sciences, Beijing 100101, China

***Correspondence to:** Junling Zhang ([junlingz@cau.edu.cn](mailto:junlingz@cau.edu.cn))

**Supplementary Table S1**

Location, vegetation composition, cultivation and soil characteristics of the three sampling sites.

|  | Site 1 | Site 2 | Site 3 |
| --- | --- | --- | --- |
| Location | Buju County | Zharao County | Juemu Village |
| Latitude | 29°28′4.6″-29°28′6.1″ N | 29°18′26.0″- 29°16′39.3″ N | 29°39′55.9″-29°40′0.8″ N |
| Longitude | 94°24′54.3″-94°25′4.8″ E | 94°18′52.7″-94°19′37.0″ E | 94°19′16.4″-94°20′12.0″ E |
| Altitude (m a.s.l.) | 2940-2965 | 2930-2957 | 3032-3096 |
| Land use types | Forest, grassland and arable land | Forest, grassland and arable land | Forest, grassland and arable land |
| Dominant species in forest | *Pinus densata*, *Picea likiangensis* var. *linzhensis*, *Quercus aquifolioides* | *Pinus densata*, *Quercus aquifolioides* | *Picea likiangensis* var. *linzhensis*, *Quercus aquifolioides, Berberis thunbergii* |
| Dominant species in grassland | *Polygonum* sp., *Aster* sp., *Geranium* sp., *Poa* spp., *Duchesnea indica, Salvia japonica, Plantago asiatica, Senecio scandens, Gueldenstaedtia verna* | *Poa* spp., *Duchesnea indica, Plantago asiatica, Gueldenstaedtia verna, Inula japonica Thunb.* | *Aster* sp., *Carum carvi*, *Potentilla* sp., *Poa* spp., *Duchesnea indica, Plantago asiatica, Senecio scandens, Gueldenstaedtia verna, Pedicularis* sp. |
| Cropping system | Winter wheat (*Triticum aestivum*) | Winter wheat (*Triticum aestivum*) | Hull-less barley (*Hordeum vulgare* var. *nudum* Hook. f.) |
| Tillage system | Conventional tillage with moldboard plow before sowing | Conventional tillage with moldboard plow before sowing | Conventional tillage with moldboard plow before sowing |
|  |  |  |  |

**Supplementary Table S2**

Chemical properties and percentages of water-stable aggregates of soils sampled from different land-use types at each sampling site.

| Land use | pH | TC  (g kg^-1^ soil) | TN  (g kg^-1^ soil) | C:N | SOM  (%) | AP  (mg kg^-1^ soil) | AN  (mg kg^-1^ soil) | Macroaggregates  (%) | Microaggregates  (%) |
| --- | --- | --- | --- | --- | --- | --- | --- | --- | --- |
| Site 1 |  |  |  |  |  |  |  |  |  |
| Forest | 5.33±0.04c | 49.9±5.7a | 4.0±0.4a | 12.53±0.31a | 6.12±1.09a | 17.9±2.6a | 8.16±1.55a | 65.7±2.2a | 30.1±2.6b |
| Grassland | 5.87±0.05b | 23.7±2.7b | 2.5±0.2b | 9.32±0.19c | 5.68±0.12a | 16.3±1.8a | 9.45±2.48a | 55.2±3.2b | 41.0±3.0a |
| Arable land | 7.81±0.09a | 15.2±0.5b | 1.4±0.1c | 10.50±0.26b | 3.57±0.32b | 16.1±0.8a | 1.30±0.48b | 52.1±2.4b | 42.1±2.6a |
| Site 2 |  |  |  |  |  |  |  |  |  |
| Forest | 5.50±0.17c | 25.6±3.7a | 2.0±0.3a | 12.68±0.42a | 5.99±0.10a | 8.0±1.0ab | 13.22±3.52a | 69.4±3.7a | 26.9±3.5b |
| Grassland | 6.15±0.06b | 23.5±5.7a | 2.0±0.4a | 11.72±0.44a | 3.37±0.88b | 5.7±0.4b | 7.73±3.00ab | 63.5±3.4a | 33.4±3.2b |
| Arable land | 7.55±0.04a | 15.6±0.7a | 1.9±0.1a | 8.16±0.15b | 2.98±1.02b | 11.5±2.3a | 3.79±1.40b | 44.5±1.6b | 52.0±1.7a |
| Site 3 |  |  |  |  |  |  |  |  |  |
| Forest | 5.41±0.08c | 24.2±3.3b | 1.7±0.2b | 14.41±0.58a | 4.49±0.97b | 15.1±3.3b | 10.91±1.30a | 74.8±2.1a | 19.6±2.1b |
| Grassland | 6.00±0.10b | 39.2±5.5a | 3.0±0.4a | 13.12±1.30a | 7.36±0.63a | 13.5±3.7b | 8.42±1.93ab | 71.6±2.3a | 24.5±2.0b |
| Arable land | 7.23±0.08a | 11.0±0.6c | 1.2±0.0b | 8.94±0.40b | 2.17±0.86b | 26.9±0.8a | 4.38±1.00b | 37.1±2.5b | 57.4±2.4a |
| Two-way ANOVA | |  |  |  |  |  |  |  |  |
| Land use | *** | *** | *** | *** | *** | ** | *** | *** | *** |
| Site | * | * | * | * | NS | *** | NS | NS | NS |
| Land use × Site | *** | *** | *** | *** | * | * | NS | *** | *** |

Data are mean ± SE (n = 5). Different lowercase letters indicate significant differences among three land use types by Duncan’s multiple range test at *p* < 0.05 level. * *p* < 0.05; ** *p* < 0.01; *** *p* < 0.001; NS, not significant.

**Supplementary Table S3**

Carbon concentration in four SOC fractions in soils of different land-use types at each sampling site. Data are mean ± SE (n = 5).

| Land use | C concentration in SOC fractions (g kg^-1^) | | | |  |
| --- | --- | --- | --- | --- | --- |
|  | Unprotected C | Physically protected C | Chemically protected C | Biochemically protected C |  |
| Site 1 |  |  |  |  |  |
| Forest | 26.27±3.29a | 10.38±1.37a | 8.95±0.17a | 0.96±0.05a |  |
| Grassland | 6.37±1.45b | 5.60±1.20b | 8.61±1.22a | 0.70±0.06b |  |
| Arable land | 1.64±0.16b | 1.48±0.08c | 8.41±0.69a | 0.65±0.02b |  |
| Site 2 |  |  |  |  |  |
| Forest | 9.06±2.29a | 4.71±0.62ab | 7.04±1.14a | 0.58±0.08a |  |
| Grassland | 8.17±3.04a | 5.16±1.12a | 6.09±1.03a | 0.63±0.10a |  |
| Arable land | 3.20±0.44a | 2.45±0.15b | 7.35±0.23a | 0.58±0.02a |  |
| Site 3 |  |  |  |  |  |
| Forest | 14.51±3.48a | 3.66±0.88b | 2.80±0.32b | 0.47±0.04b |  |
| Grassland | 19.24±3.67a | 7.50±0.84a | 7.14±1.55a | 0.96±0.21a |  |
| Arable land | 1.98±0.18b | 1.12±0.04c | 4.63±0.08ab | 0.28±0.03b |  |
| Two-way ANOVA |  |  |  |  |  |
| Land use | *** | *** | NS | ** |  |
| Site | * | * | *** | * |  |
| Land use × Site | *** | *** | * | ** |  |

Different lowercase letters indicate significant differences among three land use types by Duncan’s test at *p* < 0.05. * *p* < 0.05; ** *p* < 0.01; *** *p* < 0.001; NS, not significant.

**Supplementary Table S4**

Diversity indices of AM fungal community, hyphal length density (HLD) and glomalin contents of soils sampled from three land use types at each sampling site. Data are mean ± SE (n = 5).

| Land use | Diversity index | | | | | HLD  (m g^-1^ soil) | | Glomalin concentration | | | |  | |
| --- | --- | --- | --- | --- | --- | --- | --- | --- | --- | --- | --- | --- | --- |
|  | Richness | Shannon-Wiener | Simpson | | Evenness |  |  | T-GRSP (mg g^-1^ soil) | | EE-GRSP (mg g^-1^ soil) | |  | |
| Site 1 |  |  |  | |  |  | |  | |  | |  | |
| Forest | 23.40±2.66a | 1.54±0.24ab | 0.64±0.09ab | | 0.49±0.06ab | 45.09±8.05a | | 4.68±0.12a | | 2.48±0.06a | |  | |
| Grassland | 29.00±3.32a | 2.24±0.24a | 0.83±0.04a | | 0.66±0.05a | 39.98±13.97a | | 4.57±0.24a | | 2.12±0.15b | |  | |
| Arable land | 22.40±2.94a | 1.32±0.31b | 0.51±0.12b | | 0.42±0.08b | 16.89±0.81a | | 2.17±0.07b | | 1.00±0.07c | |  | |
| Site 2 |  |  |  | |  |  | |  | |  | |  | |
| Forest | 24.80±2.06a | 1.84±0.22a | 0.75±0.06a | | 0.57±0.05a | 32.26±4.85a | | 3.15±0.19b | | 1.96±0.12a | |  | |
| Grassland | 27.20±3.12a | 2.04±0.14a | 0.81±0.02a | | 0.62±0.02a | 28.93±7.87ab | | 4.32±0.46a | | 1.83±0.20a | |  | |
| Arable land | 20.00±2.02a | 1.64±0.22a | 0.66±0.09a | | 0.55±0.07a | 12.99±0.85b | | 2.44±0.07b | | 1.12±0.06b | |  | |
| Site 3 |  |  |  | |  |  | |  | |  | |  | |
| Forest | 19.00±1.76c | 1.33±0.24b | 0.58±0.11a | | 0.45±0.08b | 37.98±7.47a | | 3.12±0.41b | | 1.84±0.17b | |  | |
| Grassland | 39.80±2.99a | 2.36±0.20a | 0.82±0.04a | | 0.64±0.04a | 37.88±4.50a | | 4.64±0.45a | | 2.47±0.11a | |  | |
| Arable land | 29.20±3.85b | 1.80±0.21ab | 0.68±0.07a | | 0.53±0.04ab | 28.82±2.56a | | 2.18±0.06b | | 1.09±0.04c | |  | |
| Two-way ANOVA | |  | |  | | |  | |  | |  | |  |
| Land use | *** | ** | ** | | ** | ** | | *** | | *** | |  | |
| Site | NS | NS | NS | | NS | NS | | * | | NS | |  | |
| Land use×Site | * | NS | NS | | NS | NS | | ** | | *** | |  | |

Different lowercase letters indicate significant differences among the three land use types by Duncan’s multiple range test at *p* < 0.05 level. * *p* < 0.05; ** *p* < 0.01; *** *p* < 0.001; NS, not significant.

**Supplementary Table S5**

Spearman correlations between AM fungal factors (HLD and glomalin contents), percentages of soil aggregates, C and N concentrations and two major SOC fractions (a) across all land use types, (b) in forest and grassland or (c) arable land only. Boldface numbers are marginally significant at *p* < 0.1, and significant at * *p* < 0.05, ** *p* < 0.01, and *** *p* < 0.001.

|  | T-GRSP | EE-GRSP | Macroaggregates | Microaggregates | Soil TC | Soil TN | Unprotected C | Physically protected C |
| --- | --- | --- | --- | --- | --- | --- | --- | --- |
| a) All land use types |  |  |  |  |  |  |  |  |
| HLD | **0.660**** | **0.391**** | **0.439**** | **-0.436**** | **0.484**** | **0.345*** | **0.525***** | **-0.531***** |
| T-GRSP |  | **0.846***** | **0.624***** | **-0.594***** | **0.887***** | **0.808***** | **0.875***** | **0.919***** |
| EE-GRSP |  |  | **0.493**** | **-0.441**** | **0.794***** | **0.783***** | **0.750***** | **0.853***** |
| Macroaggregates |  |  |  | **-0.989***** | **0.638***** | **0.369*** | **0.705***** | **0.610***** |
| Microaggregates |  |  |  |  | **-0.613***** | **-0.335*** | **-0.677***** | **-0.573***** |
| b) Forest and grassland combined | |  |  |  |  |  |  |  |
| HLD | -0.004 | **0.514**** | 0.127 | -0.158 | **0.390*** | **0.347** | **0.391*** | **0.436*** |
| T-GRSP |  | **0.579**** | -0.242 | 0.289 | **0.536**** | **0.678***** | **0.387*** | **0.585**** |
| EE-GRSP |  |  | 0.033 | -0.031 | **0.840***** | **0.896***** | **0.712***** | **0.861***** |
| Macroaggregates |  |  |  | **-0.976***** | 0.177 | -0.068 | **0.385*** | -0.030 |
| Microaggregates |  |  |  |  | -0.159 | 0.090 | **-0.371*** | 0.051 |
| c) Arable land |  |  |  |  |  |  |  |  |
| HLD | **-0.454** | 0.082 | -0.379 | 0.325 | **-0.589*** | **-0.785**** | **-0.461** | **-0.832***** |
| T-GRSP |  | **0.632**** | 0.002 | 0.029 | **0.489** | **0.633*** | **0.500** | **0.554*** |
| EE-GRSP |  |  | -0.315 | 0.346 | 0.246 | 0.245 | **0.500** | 0.154 |
| Macroaggregates |  |  |  | **-0.963***** | **0.475** | 0.358 | -0.365 | 0.370 |
| Microaggregates |  |  |  |  | -0.421 | -0.318 | **0.514*** | -0.264 |


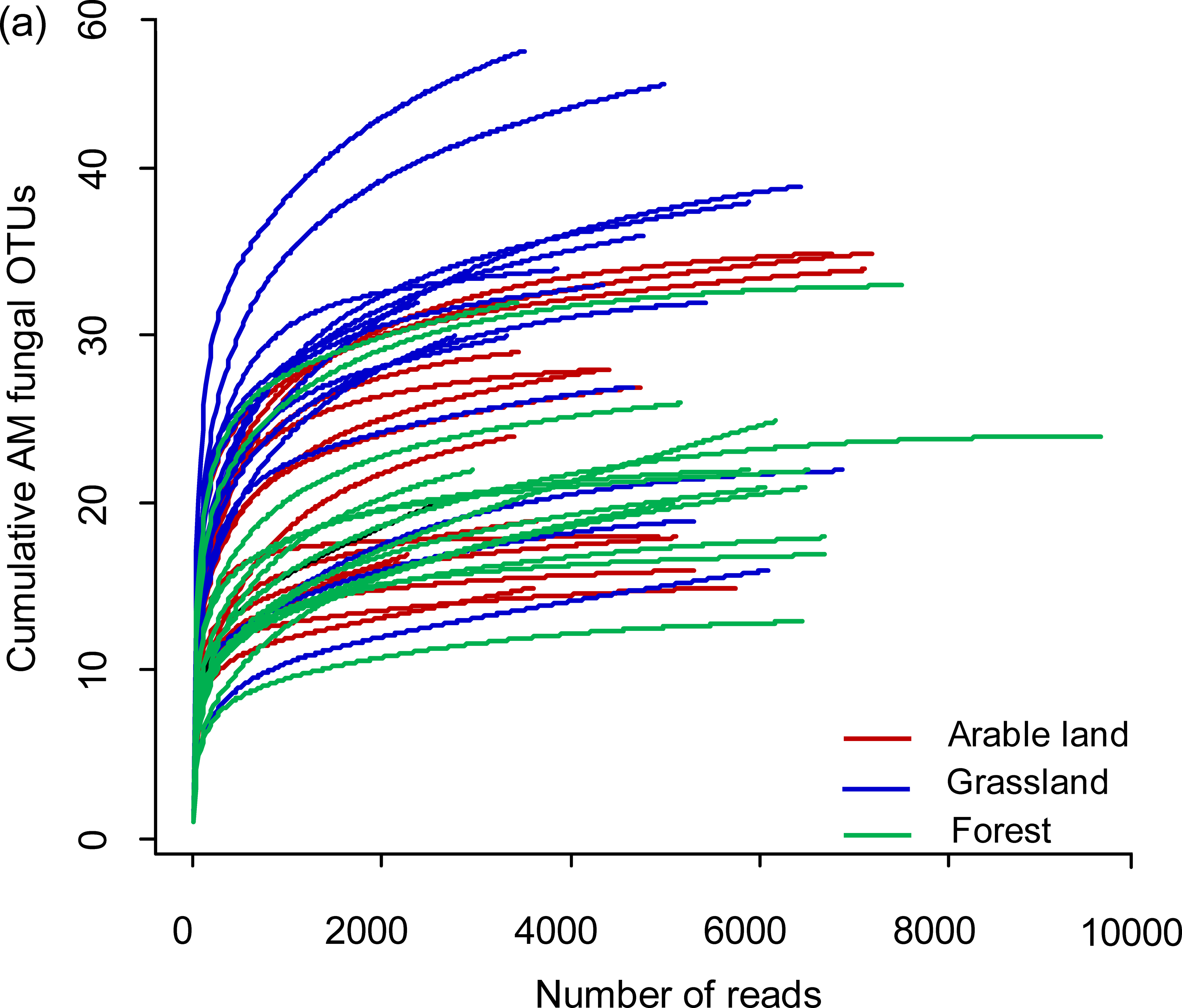

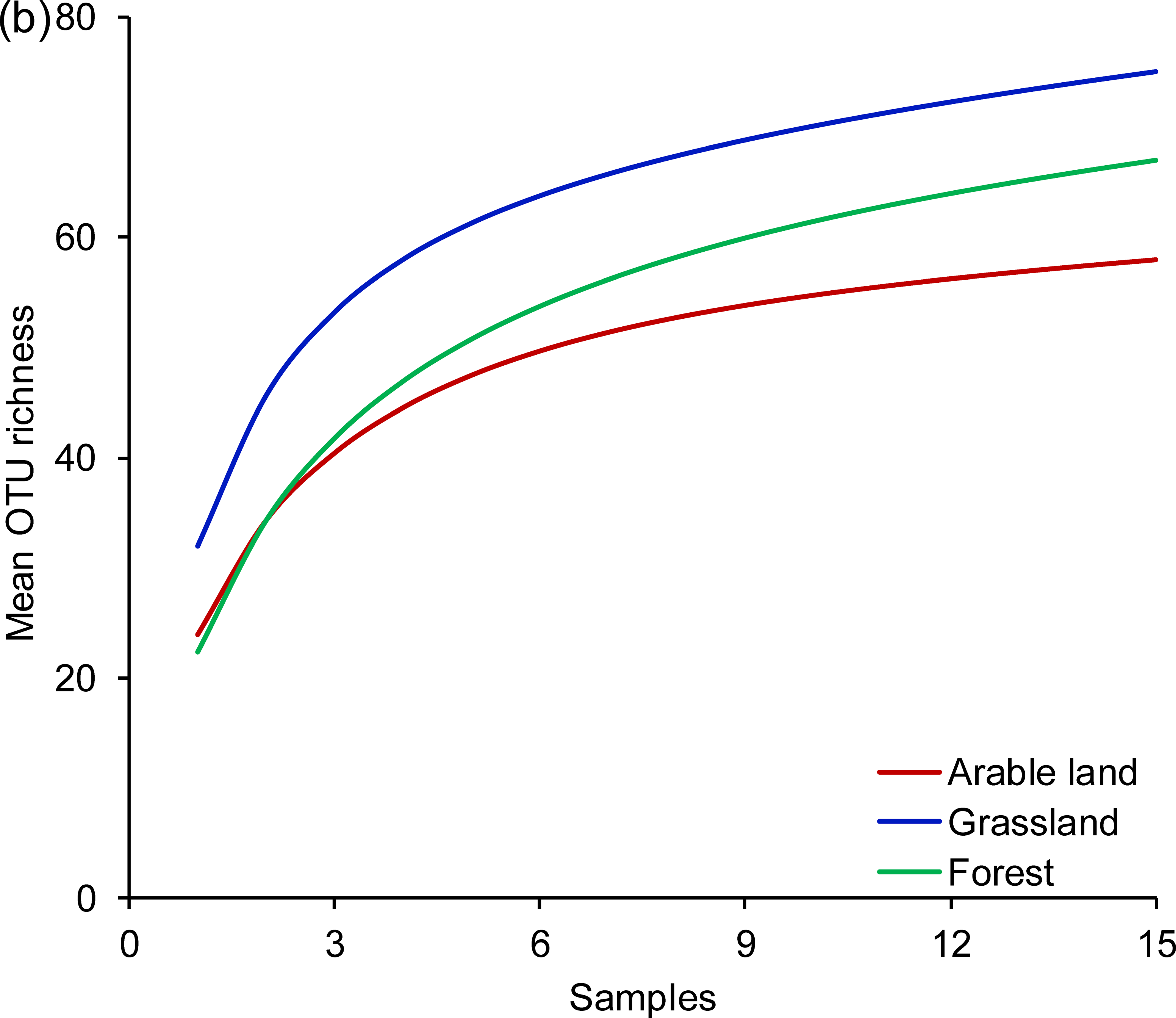


**Supplementary Fig. S1** Rarefaction analysis for observed OTUs of AM fungi based on (a) number of reads and (b) samples in the three different land use types.





**Supplementary Fig. S2** Neighbor-joining phylogram of OTUs obtained in soils sampled from forest, grassland and arable land in the study region based on the TrN+G substitution model. Numbers above the nodes indicate bootstrap support in NJ analysis. Representative sequences were selected from every OTU to structure NJ tree with PAUP 4.0. One of the most parsimonious trees is shown with bootstrap support values (1,000 replicates, > 50).


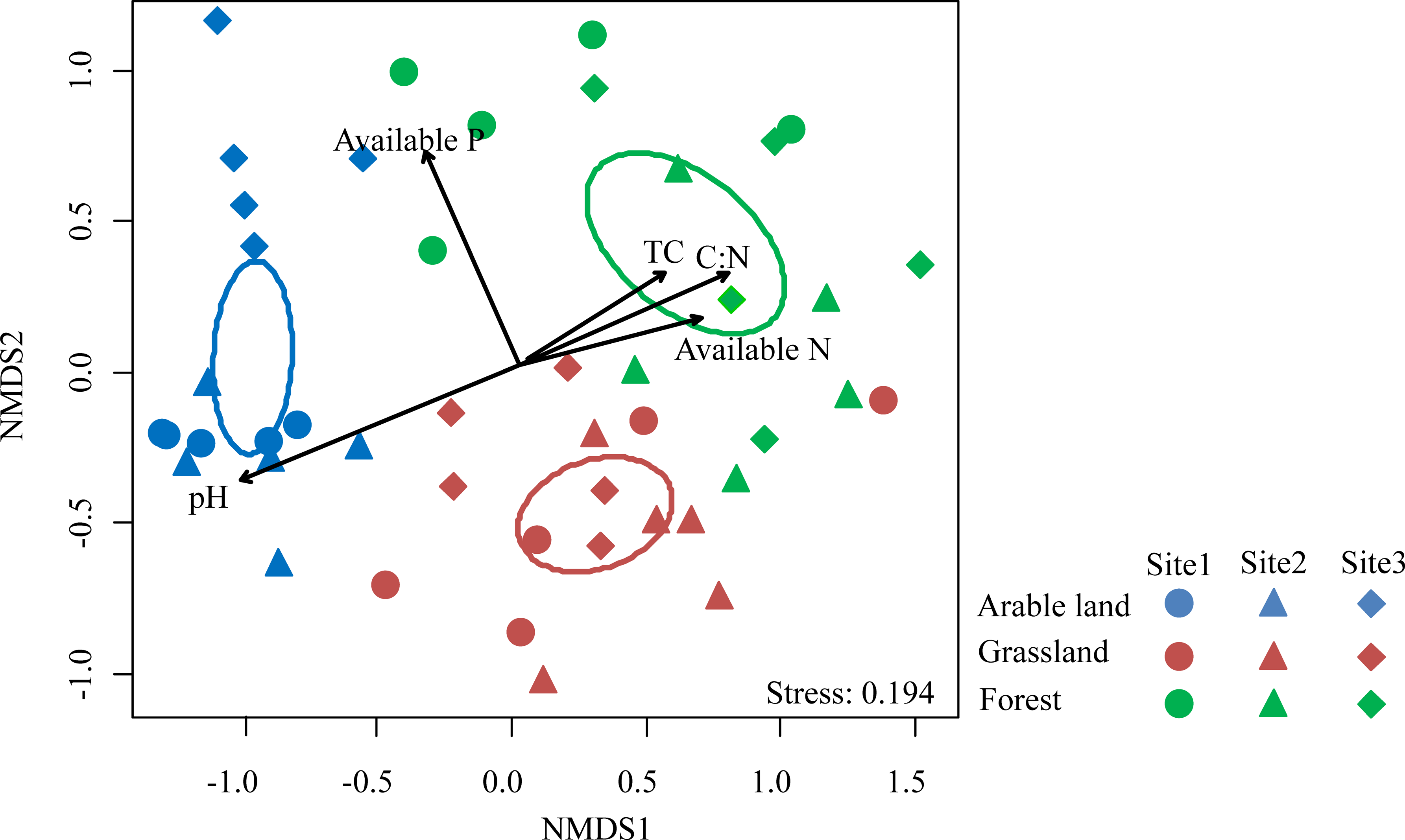


**Supplementary Fig. S3** Non-metric multidimensional scaling (NMDS) plot of AM fungal community composition and the vectors of significant environmental variables (*p* < 0.05). Different land use types and sampling sites are marked with different colors and symbols. Ellipses in the plots represent 95% confidence intervals around the average values for samples from each land use type with corresponding colors.

**
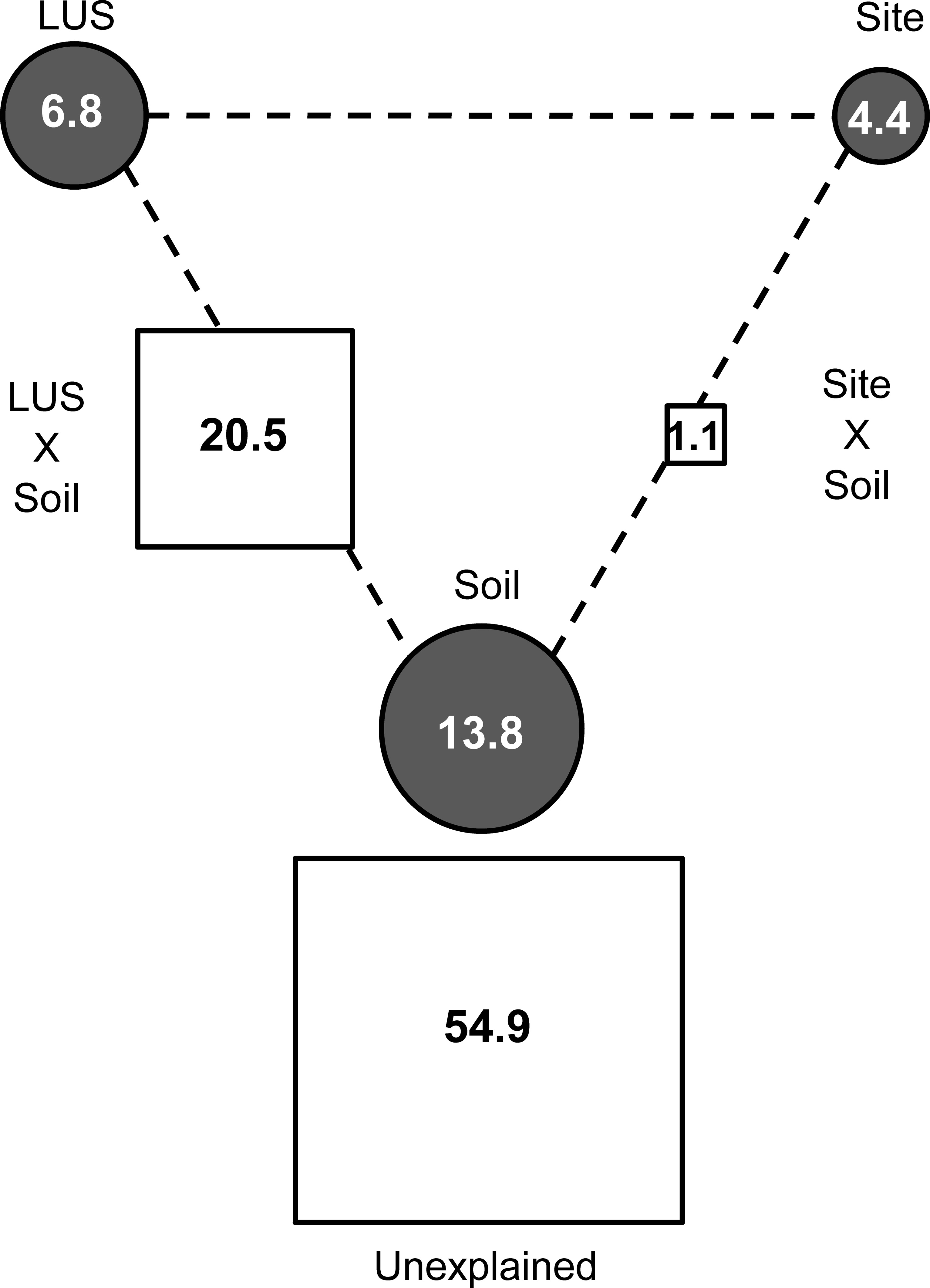
**

**Supplementary Fig. S4** Partitioning analysis of the variation in AM fungal community structure into soil properties (Soil), sampling site (Site) and land use (LUS) components. Circles on the edges of the triangle show the percentage of the variation explained by each factor alone. The joint effect between two or three of the factors is shown as squares on the sides of the triangle. The unexplained variation is depicted in the square on the bottom. The size of circles and squares is proportional to the variance explained.

**Supplementary Methods**

**Calculation of C storage**

Direct measurement of soil bulk density was not available when we sampled and the following equation was therefore use to estimate bulk density^1^:

BD = 100 / {SOM / 0.244 + (100 - SOM) / 1.64}

where BD is the estimated bulk density (g cm^-3^), SOM is the content of soil organic matter (%).

The SOC stock (Mg C ha^-1^) of the top 20 cm of the soil profile was then calculated as follows:

SOC stock = BD × C × Soil depth

where BD is the estimated bulk density, C is the measured SOC concentration (%), and soil depth is 20 cm.

By calculation with the data from the present study, the SOC stocks of forest and grassland are estimated at 73.37 Mg C ha^-1^ and 63.16 Mg C ha^-1^, respectively. The areas of forest and grassland in Nyingchi region are 2.64×10^6^ ha and 2.91×10^5^ ha, respectively. Therefore the total amount of SOC stored in forest and grassland of Nyingchi region is estimated at 0.212 Pg (73.37 Mg C ha^-1^ × 2.64 × 10^6^ ha + 63.16 Mg C ha^-1^ × 2.91 × 10^5^ ha = 0.212 × 10^9^ Mg C). The average SOC density (SOC storage per area) is then estimated at approximately 7.23 kg m^-2^, which was calculated by dividing the total SOC stock (0.212 Pg C) by the total area of forest and grassland (2.64×10^6^ ha + 2.91×10^5^ ha).

1. Post, W. M. & Kwon, K. C. Soil carbon sequestration and land-use change: Processes and potential. *Global Change Biol.* **6**, 317-327 (2000).
